# Spatiotemporal Characteristics and Influential Factors of Electronic Cigarette Web-Based Attention in Mainland China: Time Series Observational Study

**DOI:** 10.2196/66446

**Published:** 2025-02-10

**Authors:** Zhongmin Zhang, Hengyi Xu, Jing Pan, Fujian Song, Ting Chen

**Affiliations:** 1 Healthy Hubei Development and Social Progress Research Center of the Key Research Base of Humanities and Social Sciences in Hubei Province School of Public Health Wuhan University of Science and Technology Wuhan China; 2 School of Xiangtao Wuhan University of Science and Technology Wuhan China; 3 Norwich Medical School University of East Anglia Norwich United Kingdom

**Keywords:** electronic cigarettes, Baidu index, web-based attention, spatiotemporal characteristics, China

## Abstract

**Background:**

The popularity of electronic cigarettes (e-cigarettes) has steadily increased, prompting a considerable number of individuals to search for relevant information on them. Previous e-cigarette infodemiology studies have focused on assessing the quality and reliability of website content and quantifying the impact of policies. In reality, most low-income countries and low- and middle-income countries have not yet conducted e-cigarette use surveillance. Data sourced from web-based search engines related to e-cigarettes have the potential to serve as cost-effective supplementary means to traditional monitoring approaches.

**Objective:**

This study aimed to analyze the spatiotemporal distribution characteristics and associated sociodemographic factors of e-cigarette searches using trends from the Baidu search engine.

**Methods:**

The query data related to e-cigarettes for 31 provinces in mainland China were retrieved from the Baidu index database from January 1, 2015, to December 31, 2022. Concentration ratio methods and spatial autocorrelation analysis were applied to analyze the temporal aggregation and spatial aggregation of the e-cigarette Baidu index, respectively. A variance inflation factor test was performed to avoid multicollinearity. A spatial panel econometric model was developed to assess the determinants of e-cigarette web-based attention.

**Results:**

The daily average Baidu index for e-cigarettes increased from 53,234.873 in 2015 to 85,416.995 in 2021 and then declined to 52,174.906 in 2022. This index was concentrated in the southeastern coastal region, whereas the hot spot shifted to the northwestern region after adjusting for population size. Positive spatial autocorrelation existed in the per capita Baidu index of e-cigarettes from 2015 to 2022. The results of the local Moran’s *I* showed that there were mainly low-low cluster areas of the per capita Baidu index, especially in the central region. Furthermore, the male-female ratio, the proportion of high school and above education, and the per capita gross regional domestic product were positively correlated with the per capita Baidu index for e-cigarettes. A higher urbanization rate was associated with a reduced per capita Baidu index.

**Conclusions:**

With the increasing popularity of web-based searches for e-cigarettes, a targeted e-cigarette health education program for individuals in the northwest, males, rural populations, high school and above educated individuals, and high-income groups is warranted.

## Introduction

Electronic cigarettes (e-cigarettes), as emerging tobacco products, present a significantly appealing option to the public. In the European Union, 31.1% of current smokers, 10.8% of former smokers, and 2.3% of never smokers reported ever using e-cigarettes [1]. The world’s first e-cigarette was invented in Guangzhou, China. To date, China remains the principal producer of e-cigarettes, responsible for 95% of global manufacturing [2]. In 2021, China’s e-cigarette exports reached 101.5 billion RMB (US $15.91 billion; US $1=6.38 RMB), with a market size of 165.88 billion RMB (US $26 billion) [3]. Between 2015-2016 and 2018-2019, e-cigarette use among Chinese adults increased from 1.3% to 1.6% [4]. Similarly, the rate of e-cigarette use among middle school students rose from 1.2% in 2014 to 2.7% in 2019 [5].

The widespread marketing of e-cigarettes undoubtedly contributes significantly to this growth. In addition to conventional retail marketing tactics, e-cigarette manufacturers use web-based advertisement strategies to promote their merchandise [6]. On social media platforms, e-cigarettes are often portrayed as safer and healthier alternatives to traditional cigarettes [6-8]. Nonetheless, numerous studies have indicated that these products may elevate the risk of cardiovascular and respiratory diseases and adversely affect brain development and mental health [9,10]. Given the increasing prevalence of e-cigarette use and the persistent debate surrounding its health implications, an increasing number of individuals are turning to the web to access the latest information.

Web-based data offer a diverse array of perspectives and data points, with user query frequencies and concerns being well recorded. In contrast to data from traditional government communication channels, web-based data are typically anonymous, timely, and cost-effective [11,12]. Therefore, they were extensively used in disease incidence reporting, pandemic outbreak surveillance and forecasting, and public awareness analysis [13-15]. The seasonality and global nature of infectious diseases rendered them initial case studies for web-based surveillance. Tracking of influenza, HIV, and dengue outbreaks has been a continuous research hot spot [15,16]. Recently, the scope of web-based surveillance has expanded to encompass mental health, tobacco use, and noncommunicable diseases [12].

Scholarly inquiries into e-cigarette infodemiology have focused on trends in popularity, website content quality and reliability, policy impact assessments, and correlations with population use prevalence [8,17-22]. Ayers et al [17,18] used Google Trends to estimate the popularity of e-cigarettes in high-income countries such as the United States. Ghosh et al [20] suggested that web-based search data on e-cigarette–related topics in the United States and India correlate with their actual population-based prevalence. Another study conducted in the United Kingdom also confirmed this finding [19]. These studies demonstrated the great potential of using web-based search engine data to supplement and expand traditional channels for monitoring e-cigarette use. This is particularly important in low-income countries and low- and middle-income countries, where the majority have not started monitoring e-cigarette use and lack evidence to guide local policy and regulatory decisions [23,24].

To our knowledge, only 1 study has portrayed e-cigarette attention in China from 2011 to 2014 using search engine data [25]. It revealed regional differences in the Baidu search volume for e-cigarettes but did not delve into the long-term spatial and temporal evolution patterns, nor did it consider spatial effects when exploring the influencing factors. Spatiotemporal analysis can integrate multidimensional data, uncover spatiotemporal dynamic associations, and facilitate visual presentation. Previous research has used this method to explore web-based search characteristics for vaccines, migraine, and anxiety [26-28].

By December 2023, China’s web user base had reached approximately 1.1 billion [29]. Baidu dominates the search engine market with a penetration rate of 90.9% [30]. Anchored in Baidu’s vast search data, the Baidu index (BI) can quantify the search volume of specific terms in a certain geographical area within a given time range and convert it into a relative index value [31]. It largely reflects the search needs and awareness of Chinese web users and has been used for public health surveillance [26-28,31]. The purpose of this study was to use the BI to assess temporal and regional changes in web-based e-cigarette retrieval in mainland China and to identify factors associated with differences in public attention. The findings will contribute to enhancing and supplementing the traditional e-cigarette surveillance system in mainland China and provide a reference for the development and implementation of tobacco control policies and targeted health educational programs on e-cigarettes.

## Methods

### Data Sources

#### Web-Based Search Databases

The web-based search data related to e-cigarettes were derived from the official BI website. This platform allows researchers to capture search frequencies of different geographic regions using specific search terms over selected time frames. In this study, the corresponding Chinese characters for e-cigarettes and their synonyms were chosen as search terms. Considering the relatively low web-based search volume of e-cigarettes in China until 2015, the data sample period was set from January 1, 2015, to December 31, 2022. The geographical scope encompassed all 31 provinces in mainland China.

The daily average Baidu index (DBI) is the weighted sum of search frequency for search terms, calculated according to the daily search volume on Baidu. To further quantify the average number of searches per individual within each province, the per capita Baidu index (PBI) was defined as the ratio of the DBI to the year-end population.

#### Real-World Databases

To explore the influencing factors of web-based search volume, demographic and socioeconomic data were collected from the China Statistical Yearbook (2011-2022). The demographic variables included the male-female ratio, gross dependency ratio (the ratio of the nonworking-age individuals to the working-age population), and urbanization rate (the proportion of the urban population to the total population at the end of the year). The proportion of individuals with a high school education or above is the segment of the total population with at least a higher education, reflecting the educational level of the region. The per capita gross regional domestic product (GRDP), an indicator of economic development, was logarithmized in the analysis to eliminate the effects of heteroscedasticity and quantity stiffness. The descriptive statistical analysis of these variables is shown in Table S1 in Multimedia Appendix 1.

### Statistical Analysis

#### Temporal Aggregation Analysis

The concentration ratio method was applied to analyze the temporal aggregation of the DBI for e-cigarettes [32]. The formula takes the following form:



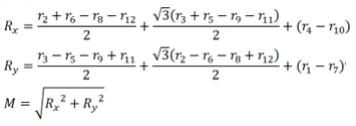



**(1)**

where*R* represents the degree of dispersion and *r*_1_indicates the ratio of searches in month to the total searches for the year. *M* shows the degree of concentration, ranging from 0 to 1. A larger value of *M* indicates a stronger temporal aggregation and a value less than 0.3 suggests a relatively uniform time distribution.

#### Spatial Weight Matrix Definition

The spatial weight matrix is the main expression of spatial adjacency relationships. Its definition is a prerequisite for conducting spatial analysis. In our study, the widely used Rook adjacency method was adopted. This method assumes that if 2 provinces have a common boundary, they are considered adjacent with a weight of 1, otherwise, it is 0 [33]. Given that Hainan Province lacks a shared border with any neighboring provinces, we defined it as adjacent to the geographically closest Guangdong Province. The definition results of the spatial matrix Rook adjacency criterion are shown in Table S2 in Multimedia Appendix 1.

#### Spatial Autocorrelation Analysis

Spatial autocorrelation analysis was used to determine the spatial aggregation of the DBI for e-cigarettes [34]. It is generally categorized into global and local spatial analysis. We used the global Moran’s I to analyze the spatial distribution pattern of the e-cigarette BI in each province of China, reflecting spatial dependence. The equation for the calculation is as follows:



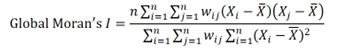



**(2)**

Where *w_ij_* represents the weight of the spatial relationship between observations *i* and *j*. The deviation of the attribute value of element *i* from its mean is denoted by . *n* is the total number of elements. The global Moran’s *I* is between –1 and 1. *I*>0 suggests the presence of a positive spatial correlation, with values closer to 1 indicating a greater degree of spatial aggregation. Conversely, *I*<0 signifies a negative spatial correlation. When *I* equals 0, it is irrelevant.

Global spatial autocorrelation analysis fails to reveal the spatial relationships and evolutionary features within a region [35]. Therefore, the local Moran’s *I* was applied to examine the correlation of provincial units and their neighboring provinces. It can identify the characteristics of aggregation and dissociation among districts. The local Moran’s *I* is defined as follows:



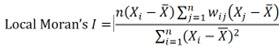



**(3)**

The indicators within the formula carry similar meanings to those in the global Moran’s *I* formula. The results of the local Moran’s *I* analysis can be categorized into 4 significant types: high-high cluster, low-low cluster, high-low outlier, and low-high outlier. Specifically, a region with high values can be identified as a high-high cluster if its neighboring regions also have high values. If a regional unit with low values is surrounded by other regional units with low values, that unit may be classified as a low-value cluster. High-low outliers denote high-attention regions encircled by low-attention regions, whereas low-high outliers indicate low-attention regions surrounded by high-attention regions. The global and local Moran’s *I* were computed using GeoDa 12.0 (Dr Luc Anselin’s team). Regional distribution maps were drawn with ArcGIS 10.8 (Environmental Systems Research Institute).

#### Spatial Panel Econometric Model Description

Unlike traditional panel regression models, spatial panel econometric models consider the spatial correlation and heterogeneity among regions [36]. The classical spatial panel econometric analysis models include the spatial autoregressive regression model (SAR), spatial error model (SEM), and spatial Durbin model (SDM). These models differ in their assumptions regarding spatial interaction. SAR solely incorporates the spatial dependence of explanatory variables, SEM focuses exclusively on the spatial dependence of the random error term, and SDM integrates both effects. The basic formulas are defined as follows:



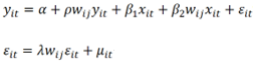



**(4)**

Where *y_it_* is the dependent variable of province *i* at time *t*. *x* is the explanatory variable. α represents the intercept item. ρ is the spatial autocorrelation coefficient and *w_ij_* is the spatial weight matrix. β is the vector of explanatory power, ε is the residual disturbance term, and λ is the spatial error coefficient. When λ=0 and β_2_=0, the above model can directly degenerate into the SAR; when ρ=0 and β_2_=0, the model can evolve into the SEM and when λ=0, the model transforms into the SDM.

#### Selection of a Suitable Spatial Panel Econometric Model

The Lagrange multiplier test was used to identify the presence of spatial effect [37]. The Wald test and likelihood ratio (LR) test were performed to evaluate whether SDM could be degraded into the SAR or SEM. The Hausman test was used to compare the suitability of fixed and random effects [38]. The LR time-space effect test was finally applied to determine the type of spatial effect. The selection process is shown in Figure S1 in Multimedia Appendix 1.

Table S3 in Multimedia Appendix 1 shows the results of the spatial panel model test. The Lagrange multiplier test suggested that the SDM was more appropriate than the SEM and the SAR. The Wald test and the LR test further confirmed that the SDM could not be degraded into the SAR and the SEM. The estimated values of the Hausman test rejected the null hypothesis, indicating that fixed effect was better than random effect in this study. In addition, the time-space double effect is more suitable for objective reality, as evidenced by the significant LR test results (LR-both/space=53.780; *P*<.001; LR-both/time=777.650; *P*<.001). Consequently, a time-space double fixed effects SDM was used to analyze the influencing factors of PBI for e-cigarettes. All tests were conducted using Stata/MP 16 (StataCorp). A 2-sided *P* value of <.1 was considered to indicate statistical significance.

#### Multicollinearity Test

Multicollinearity refers to the linear relationship between 2 or more independent variables. To address potential multicollinearity problems in this study, we conducted a variance inflation factor test. The results are shown in Table S4 in Multimedia Appendix 1. Notably, the variance inflation factor values for all variables did not exceed 10, indicating the absence of multicollinearity in our model.

### Ethical Considerations

Data for this study were obtained from publicly available BI search data and statistical yearbooks. No human or animal subjects were involved. Data privacy and security were approved by the ethics committee of Wuhan University of Science and Technology (approval number 202 063).

## Results

### Temporal Characteristics

**Figure 1:**
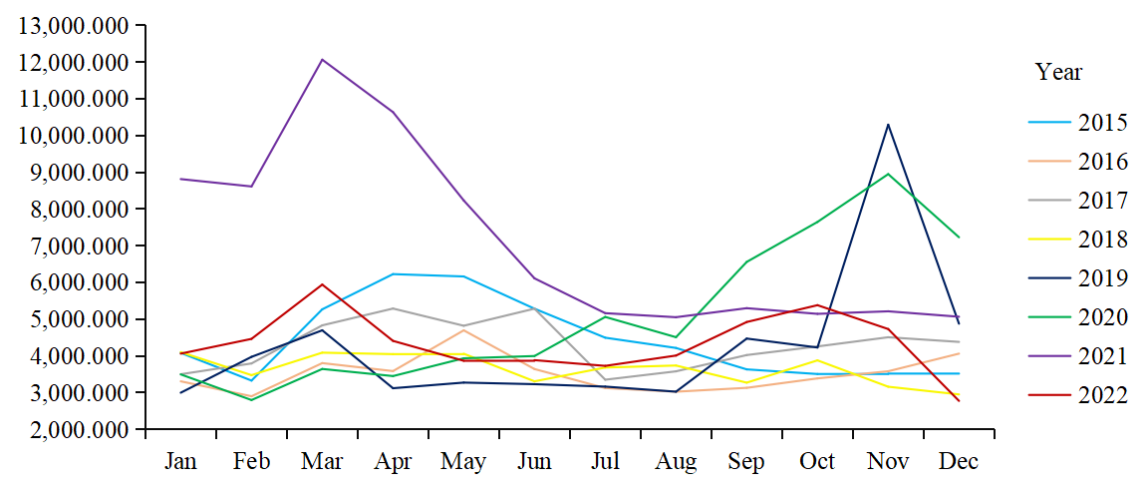
Seasonal plot of the daily average Baidu index for electronic cigarettes (2015-2022).

Table S5 in Multimedia Appendix 1 and Figure 1 show the temporal distribution characteristics of the DBI for e-cigarettes from January 2015 to December 2022. Over the 8-year period, the average DBI was 55,137.045. Generally, the DBI for e-cigarettes follows a fluctuating yet upward trajectory. The highest increase occurred in 2020-2021, with the DBI rising from 61,277.286 to 85,416.995. In 2022, the DBI decreased to 52,174.906. During the study period, the concentration ratios of DBI varied between 0.136 and 0.417. Specifically, the maximum values were recorded in 2019 (*M*=0.417) and 2020 (*M*=0.399). In both of these years, the peak DBI values appeared in October, November, and December.

### Spatial Characteristics

**Figure 2:**
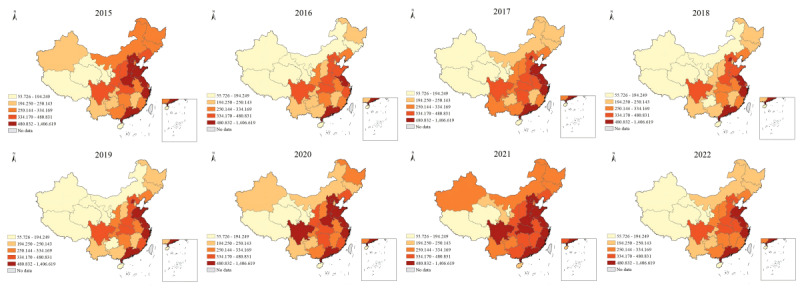
The daily average Baidu index of electronic cigarettes by province in China (2015-2022).

**Figure 3:**
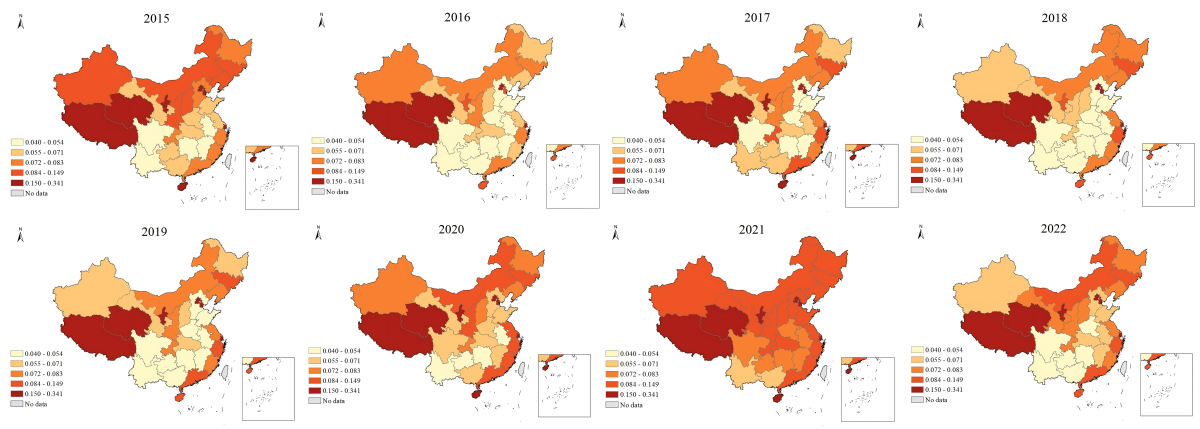
The per capita Baidu index of electronic cigarettes by province in China (2015-2022).

Figures 2 and 3 depict the DBI and the PBI of e-cigarettes in each Chinese province from 2015 to 2022, respectively. Provinces with higher DBI for e-cigarettes include Shandong, Jiangsu, Zhejiang, and Guangdong, all located on the southeast coast of China. These regions consistently rank at the top, with annual DBI exceeding 450. Qinghai, Tibet, and Ningxia maintained the lowest DBI levels. The geographical pattern of the PBI contrasts significantly with that of the DBI, as the former is concentrated in Tibet, Qinghai, and Ningxia. Notably, the PBI for e-cigarettes in Beijing far surpassed that in neighboring provinces.

Table 1 indicates the global Moran’s *I* of the PBI for e-cigarettes from 2015 to 2022. Throughout the period, all years had a global Moran’s *I* value greater than 0, ranging between 0.151 and 0.265. The *z* scores revealed that spatial clustering of the PBI for e-cigarettes was statistically significant. Despite fluctuations in the global Moran’s *I* and *z* values, the clustering pattern and positive spatial autocorrelation remained stable. The local Moran’s *I* findings for the PBI of e-cigarettes are detailed in Table 2. Low-low clusters exist in the central region of China. Guizhou and Hubei consistently demonstrated significant low-low clusters over the 8-year period. Tianjin displayed a high-high cluster only in 2015. Furthermore, Xinjiang, located in the northwestern region, was identified as a low-high outlier area in 2016, 2017, and 2022.

**Table 1:** The global Moran’s I of the per capita Baidu index for electronic cigarettes (2015-2022)^a^.

Year	Global Moran’s *I*	*z* value	*P* value
2015	0.265	2.612	.009
2016	0.155	1.574	.07
2017	0.151	1.536	.08
2018	0.196	1.999	.04
2019	0.155	1.566	.07
2020	0.186	1.884	.04
2021	0.174	1.805	.05
2022	0.166	1.692	.06

^a^Significance levels were determined by *P* value tests of standardized *z* values.

**Table 2:** Local spatial aggregation of the per capita Baidu index for electronic cigarettes (2015-2022)^a^.

Year	High-high cluster	High-low outlier	Low-high outlier	Low-low cluster
2015	Tianjin	—^b^	Hebei	Guizhou, Anhui, Chongqing, Jiangxi, Hubei, Hunan, and Guangxi
2016	—	—	Xinjiang	Guizhou, Anhui, Hubei, and Shandong
2017	—	—	Xinjiang	Guizhou, Chongqing, Hubei, Shandong, and Henan
2018	—	—	—	Guizhou, Chongqing, Hubei, Hunan, Henan, and Guangxi
2019	—	—	—	Guizhou, Chongqing, Hubei, Shandong, and Henan
2020	—	—	—	Guizhou, Chongqing, Hubei, Hunan, and Guangxi
2021	—	—	—	Guizhou, Chongqing, Hubei, Hunan, and Guangxi
2022	—	—	Xinjiang	Guizhou, Chongqing, Hubei, Hunan, Henan, and Guangxi

^a^Provinces with nonsignificant local Moran’s *I* are not listed.

^b^Not available.

### Influencing Factors

Table 3 illustrates the SDM results of the PBI for e-cigarettes. The male-female ratio, the proportion of high school and above education, and the GRDP were positively correlated with the PBI for e-cigarettes. A higher urbanization rate was associated with a reduced PBI. The PBI for e-cigarettes had significant positive spatial spillover effects (spatial ρ=0.467; *P*<.001). The proportion of high school and above education significantly impacted the PBI for e-cigarettes in adjacent proximity. To further analyze these spillover effects, the SDM was decomposed into direct and indirect effects using partial differential decomposition. In terms of the direct effect, there was a significant association between the PBI and male-female ratio, urbanization rate, and GRDP in the local region. The indirect effects reveal a significant negative association between the proportion of high school education and above and the PBI for e-cigarettes in neighboring areas. Moreover, both the urbanization rate and the proportion of high school and above education negatively influence the PBI of e-cigarettes across all areas.

**Table 3:** Spatial Durbin model results of the per capita Baidu index for electronic cigarettes.

		Coefficient				95% CI				*P* value			
**Main^a^**													
	Male-female ratio				0.057				0.008 to 0.106				.02
	Gross dependency ratio				–0.002				–0.071 to 0.067				.96
	Urbanization rate				–0.145				–0.282 to –0.009				.04
	Proportion of education at high school and above				0.069				0.002 to 0.137				.04
	Ln (per capita gross regional domestic product)^b^				0.017				0.001 to 0.032				.03
**Wx^c^**													
	Male-female ratio		0.022				–0.077 to 0.122				.66		
	Gross dependency ratio	–0.042				–0.181 to 0.098				.56			
	Urbanization rate	–0.094				–0.381 to 0.193				.52			
	Proportion of education at high school and above	–0.203				–0.342 to –0.064				.004			
	Ln (per capita gross regional domestic product)	–0.009				–0.033 to 0.016				.49			
Spatial ρ^d^		0.467				0.333 to 0.602				<.001			
**Direct effect^e^**													
	Male-female ratio			0.065				0.008 to 0.122				.03	
	Gross dependency ratio			–0.008				–0.08 to 0.064				.82	
	Urbanization rate			–0.161				–0.287 to –0.035				.01	
	Proportion of education at high school and above			0.046				–0.021 to 0.114				.18	
	Ln (per capita gross regional domestic product)			0.016				0.001 to 0.032				.03	
**Indirect effect^f^**													
	Male-female ratio		0.090				–0.094 to 0.274				.34		
	Gross dependency ratio	–0.070				–0.321 to 0.181				.59			
	Urbanization rate	–0.295				–0.756 to 0.167				.21			
	Proportion of education at high school and above	–0.288				–0.523 to –0.053				.02			
	Ln (per capita gross regional domestic product)	–0.001				–0.04 to 0.037				.94			
**Total effect^g^**													
	Male-female ratio	0.155				–0.067 to 0.376				.17			
	Gross dependency ratio	–0.078				–0.365 to 0.209				.59			
	Urbanization rate	–0.456				–0.938 to 0.026				.06			
	Proportion of education at high school and above	–0.242				–0.503 to 0.02				.07			
	Ln (per capita gross regional domestic product)	0.015				–0.029 to 0.059				.50			

^a^Main denotes the impact coefficient of the variable on the local region.

^b^Ln (per capita gross regional domestic product) indicates the logarithm of per capita gross regional domestic product.

^c^Wx refers to the spatial spillover coefficient of the variable to adjacent regions.

^d^Spatial ρ represents spatial autocorrelation coefficients.

^e^Direct effect is the influence of the change in the local independent variable on the local dependent variable.

^f^Indirect effect is the influence of the change in the local independent variable on the adjacent region.

^g^The coefficient of total effects is the sum of direct and indirect effects.

## Discussion

### Principal Results and Comparison With Prior Work

In this study, we used the BI to explore the spatial and temporal distribution of web-based searches for e-cigarettes in mainland China from 2015 to 2022 and identified the complex associations between the e-cigarette searches and demographic characteristics, economic development level, and education attainment.

The upward trend in e-cigarette–related BI observed in our study aligns with evidence from high-income countries [17,18]. This may be attributed to the increasing prevalence of e-cigarette usage, improved health literacy, and increased web penetration [4,29,39]. Notably, a pronounced spike in search queries was detected in November 2019. A possible explanation for this temporary surge is “policy shock” [22,40]. At the beginning of that month, the Chinese government successively issued the “Notice on Further Protection of Minors from E-cigarettes” and the “Notice on Further Strengthening Tobacco Control Work Among Young People.” These 2 notices explicitly prohibited manufacturers from advertising and retailers from selling e-cigarettes on the web to minors [41,42]. This prompted the public and the media to focus on web-based information about e-cigarettes. In addition, the COVID-19 pandemic might be a crucial factor for the rise in e-cigarette web-based information-seeking behavior in 2019 and 2020. The implementation of stay-at-home orders and temporary retail closures fostered an environment conducive to heightened web-based engagement [43]. During this period, 18.2%-19.8% of e-cigarette users shifted their purchasing habits from retail to web-based stores [44].

In the spatial dimension, significant regional differences exist in e-cigarette web-based attention across provinces in mainland China. The DBI for e-cigarettes is high in eastern coastal regions. Although national epidemiological data on e-cigarettes are lacking, this geographic distribution may reflect the socioeconomic and population conditions. These eastern coastal regions are characterized by dense populations and well-developed economies [45]. Residents here boasted better web-based access and public health awareness [27,31]. Among the provinces, Guangdong holds the highest DBI rankings for e-cigarettes [46]. Besides demographic and economic considerations, Guangdong was the pioneer manufacturing and production base for e-cigarettes in China and the world [47]. Regarding provinces with higher PBI, Tibet and Qinghai stand out. These 2 western provinces are distinguished by their high altitudes and unique climatic conditions In such environments, traditional tobacco combustion may be impeded. E-cigarettes show greater adaptability due to their closed system and ignition-free operation [48]. It is noteworthy that Guizhou and Hubei Province in the central region were identified as a significant low-low cluster over the 8-year period. These are both large traditional tobacco-consuming provinces, where smokers may have deep-rooted consumption habits of traditional cigarettes and are been less receptive to emerging e-cigarette products [49].

### The Influencing Factors of E-Cigarette Web-Based Attention Are Diverse

First, males reported a greater frequency of e-cigarette web-based inquiries than females did. A study indicated that the proportion of males who had used and heard of e-cigarettes surpassed that of females in China [50]. Meanwhile, males are more likely to obtain e-cigarette information from online platforms and forums [51].

Second, we found a positive correlation between the ratio of high school and above education and the e-cigarette PBI. The previous study has also revealed that a greater percentage of individuals with a higher level of education had both used and been informed about e-cigarettes than did those with lower educational attainment [50]. An individual’s education level is a critical determinant of public health information literacy [52]. With the accumulation of knowledge, the understanding of e-cigarette potential harm has gradually increased. Furthermore, highly educated groups tend to be more socially active and susceptible to peer pressure.

As expected, GRDP was significantly positively correlated with web-based attention. This finding was in line with the results on regional differences. The growth of GRDP is often accompanied by improved infrastructure, comprehensive services, and increased digitalization [26,27].

However, our study found a negative correlation between the urbanization rate and the PBI of e-cigarettes. On the one hand, in highly urbanized cities, such as Beijing and Shanghai, the prohibition of smoking in public places was effectively enforceable [53]. On the other hand, in regions with higher urbanization rates, consumers’ awareness of e-cigarettes may have matured and their preferences have been largely established. New users’ curiosity-driven search behavior was substantially reduced. Moreover, there are more e-cigarette retail shops in these areas. Consumers are more likely to have on-site experiences instead of conducting web-based searches.

### Limitations

Although this study provided valuable evidence on the status and trends of e-cigarette popularity in China, there are still several limitations. First, web-based search query activities are inherently biased toward groups with higher socioeconomic levels and educational attainment. It fails to capture some important populations for e-cigarette use, especially adolescents. Second, the data available on the BI platform are limited to search terms that meet established user access thresholds. As a result, less common or infrequently used expressions may be excluded from trend analysis. Third, even though Baidu is the primary search engine among Chinese users, a minority still use alternative searches such as Google and Bing. Our study did not cover these populations. Finally, the BI data we used were collected at the provincial level. Further research should consider smaller geographic units to obtain more accurate evidence.

### Conclusions

Public interest in e-cigarettes is increasing in China. There are notable differences in the PBI for e-cigarettes among provinces, with northwest and economically prosperous coastal areas becoming hot spots. In addition, the male-female ratio, urbanization ratio, education attainment, and level of regional economic development were important factors contributing to the web-based attention to e-cigarettes. These findings underscore the necessity of devising targeted e-cigarette health education programs for individuals in the northwest, males, rural populations, high school and above educated individuals, and those with higher incomes. For less concerned groups, the focus should be on educating them about the accuracy of the information they access, thereby preventing the spread of misinformation. Our study not only contributes to supplementing and enhancing the e-cigarette use monitoring systems in China but also provides a valuable reference method for other low-income countries and low- and middle-income countries with similar patterns of web use.

## Appendix

Multimedia Appendix 1 Supplementary figures and tables on the methodology and results in the manuscript.

## Abbreviations

BI: Baidu index
DBI: daily average Baidu index
e-cigarette: electronic cigarette
GRDP: per capita gross regional domestic product
HIC: high-income country
LR: likelihood ratio
PBI: per capita Baidu index
SAR: spatial autoregressive regression model
SDM: spatial Durbin model
SEM: spatial error model
