# Supplementary material for: Spatiotemporal Characteristics and Influential Factors of Electronic Cigarette Web-Based Attention in Mainland China: Time Series Observational Study
Source: J Med Internet Res. 2025 Feb 10;27:e66446. doi: 10.2196/66446 (PMC11851045; doi:10.2196/66446)
Supplement: Multimedia Appendix 1 [file jmir_v27i1e66446_app1.docx]

# Multimedia Appendix 1

**Table S1** Descriptive statistics of the explanatory variables

| Variable | Unit | Mean | SD | Min | Max |
| --- | --- | --- | --- | --- | --- |
| Male-female ratio | % | 1.045 | 0.042 | 0.956 | 1.232 |
| Gross dependency ratio | % | 0.409 | 0.070 | 0.257 | 0.578 |
| Urbanization rate | % | 0.617 | 0.117 | 0.289 | 0.893 |
| Proportion of education at high school and above | % | 0.329 | 0.099 | 0.111 | 0.816 |
| Per Capita Gross Regional Domestic product | thousand Yuan | 67.335 | 31.378 | 26.914 | 190.526 |

**Table S2** The definition results of the spatial matrix Rook adjacency criterion

| The number of neighboring provinces | Province |
| --- | --- |
| 1 | Hainan |
| 2 | Beijing, Tianjin, Shanghai, and Heilongjiang |
| 3 | Liaoning, Jilin, Fujian, Ningxia, and Xinjiang |
| 4 | Shanxi, Tibet, Shandong, Jiangsu, Yunnan, Guangxi, and Qinghai |
| 5 | Chongqing, Zhejiang, Guangdong, and Guizhou |
| 6 | Hubei, Hunan, Henan, Anhui, Jiangxi, and Gansu |
| 7 | Hebei, and Sichuan |
| 8 | Shaanxi, and Inner Mongolia |

**Figure S1** Flow diagram of spatial econometric model selection

**Table S3** Results of the spatial panel econometric model selection test

| Spatial panel model test | | Statistic value | *P* |
| --- | --- | --- | --- |
| LM test | Moran's I | 2.970 | 0.003 |
|  | LM-error | 7.190 | 0.007 |
|  | Robust-LM-error | 2.557 | 0.11 |
|  | LM-lag | 13.214 | <0.001 |
|  | Robust-LM-lag | 8.582 | 0.003 |
| Hausman test | / | 48.840 | <0.001 |
| LR test | LR-SDM/SAR | 29.280 | <0.001 |
|  | LR-SDM/SEM | 33.910 | <0.001 |
| Wald test | Wald-SDM/SAR | 18.410 | 0.002 |
|  | Wald-SDM/SEM | 12.590 | 0.03 |
| LR test | LR-both/space | 53.780 | <0.001 |
|  | LR-both/time | 777.650 | <0.001 |

**Table S4** Results of variance inflation factor test for explanatory variables

| Variable | VIF |
| --- | --- |
| Male-female ratio | 1.13 |
| Gross dependency ratio | 1.45 |
| Urbanization rate | 7.17 |
| Proportion of education at high school and above | 4.52 |
| Per Capita Gross Regional Domestic product | 4.59 |

**Table S5** The concentration ratio of the daily average Baidu index for electronic cigarettes, 2015-2022

| Year | January | February | March | April | May | June | July | August | September | October | November | December | M^a^ |
| --- | --- | --- | --- | --- | --- | --- | --- | --- | --- | --- | --- | --- | --- |
| 2015 | 4,082.871 | 3,328.071 | 5,268.194 | 6,226.600 | 6,163.161 | 5,285.267 | 4,495.516 | 4,215.968 | 3,632.867 | 3,506.774 | 3,518.100 | 3,511.484 | 0.319 |
| 2016 | 3,307.742 | 2,901.103 | 3,803.774 | 3,586.267 | 4,696.194 | 3,641.967 | 3,124.613 | 3,025.226 | 3,130.267 | 3,387.516 | 3,582.033 | 4,061.323 | 0.246 |
| 2017 | 3,500.677 | 3,794.679 | 4,831.871 | 5,289.533 | 4,820.742 | 5,286.633 | 3,351.677 | 3,579.806 | 4,022.933 | 4,257.548 | 4,507.700 | 4,381.000 | 0.240 |
| 2018 | 4,101.774 | 3,476.071 | 4,088.000 | 4,047.967 | 4,059.452 | 3,307.833 | 3,683.065 | 3,738.129 | 3,271.533 | 3,878.452 | 3,160.633 | 2,951.290 | 0.229 |
| 2019 | 2,997.645 | 3,973.929 | 4,697.065 | 3,119.700 | 3,273.452 | 3,230.133 | 3,163.677 | 3,026.000 | 4,471.867 | 4,228.774 | 1,0295.167 | 4,877.871 | 0.417 |
| 2020 | 3,496.161 | 2,797.931 | 3,644.258 | 3,450.833 | 3,935.032 | 3,996.200 | 5,060.935 | 4,509.161 | 6,559.133 | 7,647.161 | 8,949.933 | 7,230.548 | 0.399 |
| 2021 | 8,814.677 | 8,613.929 | 1,2066.065 | 1,0637.167 | 8,232.258 | 6,111.600 | 5,163.419 | 5,053.323 | 5,299.133 | 5,144.968 | 5,214.133 | 5,066.323 | 0.200 |
| 2022 | 4,062.000 | 4,463.536 | 5,942.065 | 4,408.800 | 3,867.000 | 3,884.133 | 3,728.452 | 4,009.258 | 4,922.833 | 5,381.129 | 4,730.700 | 2,775.000 | 0.136 |

^a^The calculation method of M can be found in the temporal aggregation analysis section.
